# The Relationship between Dyslipidemia and Acute Axonal Function in Type 2 Diabetes Mellitus *In Vivo*

**DOI:** 10.1371/journal.pone.0153389

**Published:** 2016-04-14

**Authors:** Natalie C. G. Kwai, William Nigole, Ann M. Poynten, Christopher Brown, Arun V. Krishnan

**Affiliations:** 1 Prince of Wales Clinical School, University of New South Wales, Sydney, Australia; 2 Department of Endocrinology, Prince of Wales Hospital, Sydney, Australia; 3 National Health and Medical Research Council Clinical Trials Centre, University of Sydney, Sydney, Australia; University of Lancaster, UNITED KINGDOM

## Abstract

**Objectives:**

Diabetic peripheral neuropathy (DPN) is a common and debilitating complication of diabetes mellitus. Treatment largely consists of symptom alleviation and there is a need to identify therapeutic targets for prevention and treatment of DPN. The objective of this study was to utilise novel neurophysiological techniques to investigate axonal function in patients with type 2 diabetes and to prospectively determine their relationship to serum lipids in type 2 diabetic patients.

**Methods:**

Seventy-one patients with type 2 diabetes were consecutively recruited and tested. All patients underwent thorough clinical neurological assessments including nerve conduction studies, and median motor axonal excitability studies. Studies were also undertaken in age matched normal control subjects(n = 42). Biochemical studies, including serum lipid levels were obtained in all patients. Patient excitability data was compared to control data and linear regression analysis was performed to determine the relationship between serum triglycerides and low density lipoproteins and excitability parameters typically abnormal in type 2 diabetic patients.

**Results:**

Patient mean age was 64.2±2.3 years, mean glycosylated haemoglobin (HbA1c%) was 7.8±0.3%, mean triglyceride concentration was 1.6±0.1 mmol/L and mean cholesterol concentration was 4.1±0.2mmol/L. Compared to age matched controls, median motor axonal excitability studies indicated axonal dysfunction in type 2 diabetic patients as a whole (T2DM) and in a subgroup of the patients without DPN (T2DM-NN). These included reduced percentage threshold change during threshold electrotonus at 10–20ms depolarising currents (TEd10–20ms)(controls 68.4±0.8, T2DM63.9±0.8, T2DM-NN64.8±1.6%,*P*<0.05) and superexcitability during the recovery cycle (controls-22.5±0.9, T2DM-17.5±0.8, T2DM-NN-17.3±1.6%,*P*<0.05). Linear regression analysis revealed no associations between changes in axonal function and either serum triglyceride or low density lipoprotein concentration when adjusted for renal function, a separate risk factor for neuropathy development. Our findings indicate that acutely, serum lipids do not exert an acute effect on axonal function in type 2 diabetic patients: TEd(10–20ms)(1.2(-1.4,3.8);*P* = 0.4) and superexcitability (2.4(-0.05, 4.8);*P* = 0.06).

**Conclusions:**

These findings suggest that serum triglyceride levels are not related to axonal function in type 2 diabetic patients. Additional pathogenic mechanisms may play a more substantial role in axonal dysfunction prior to DPN development.

## Introduction

Diabetes mellitus is frequently complicated by the development of a length-dependent peripheral neuropathy (DPN). DPN is characterised by debilitating symptoms such as pain, paraesthesia and burning which can lead to reduced quality of life [1, 2]. Currently there is no cure and management primarily consists of symptom relief [3]. There is therefore a need for early diagnosis and identification of etiological factors which underlie DPN development and that can provide avenues for preventative care.

Traditionally, emphasis has been placed on hyperglycaemia as the primary etiological factor in DPN development. However, clinical trials administering stringent glucose control regimens have yielded disparate outcomes in terms of DPN development between type 1 and type 2 diabetic patients [4–7]. These findings have alluded to differential mechanisms of neuropathy development between type 1 and type 2 diabetes [8]. Specifically, type 2 diabetes often emerges in the setting of the metabolic syndrome, and thus the pathogenesis of DPN development in this cohort may be influenced by features of metabolic syndrome that are otherwise not present in type 1 diabetes [9]. Dyslipidemia is a prominent feature in the type 2 diabetic population and large epidemiological studies have implicated hyperlipidemia as a predictor of more severe neuropathy [10] although the mechanisms are still not well understood.

Previous studies in type 2 diabetic patients have revealed alterations in peripheral axonal function using axonal excitability techniques, which provide information on the behaviour of axonal ion channels and energy-dependent pumps and exchangers [11]. Previous studies in DPN have demonstrated changes in excitability parameters possibly due to dysfunction of the energy-dependent Na^+^/K^+^ pump [12–14]. The underlying basis for the alterations in axonal excitability in DPN patients remains unclear and previous investigations have suggested possible associations with estimated glomerular filtration rates (eGFR) [12, 15, 16], HbA1c% [15–18] and serum triglyceride levels [16]. However, the majority of these investigations were undertaken in small cohorts and changes were evaluated using post-hoc analysis rather than prospectively. Furthermore, association between excitability changes and biochemical parameters did not account for potential confounders such as baseline neuropathy severity and alterations in renal function [11].

Therefore the aim of the present study was to prospectively assess the potential contribution of serum metabolic parameters, specifically triglyceride and low-density-lipoprotein (LDL) levels, to altered excitability profiles in patients with type 2 diabetes. Statistical analysis was undertaken using linear regression, adjusted for renal function and severity of existing neuropathy.

## Research Design and Methods

Clinical neurological assessments and axonal excitability studies were conducted in 98 consecutive type 2 diabetic patients recruited from the Diabetes Centre at Prince of Wales Hospital in Sydney. Patients who were receiving neuropathic pain treatment or who had a prior history of neuropathy due to other causes were excluded from the study. A total of 71 type 2 diabetic patients were subsequently enrolled. These analyses were conducted in R (Version 3.1.0) by a biostatistician (C.B.). While no specific power calculation was undertaken, the sample size was based on a previous study of 30 diabetic subjects by Bae and colleagues which demonstrated an association between triglycerides and axonal function on post-hoc analysis but which did not control for changes in renal function [16]. All subjects gave written informed consent in accordance with the Declaration of Helsinki, and the study was approved by the South Eastern Sydney Area Health Service and the University of New South Wales Research Ethics committees (HREC#14/012). Pathology studies were undertaken at the South Eastern Area Laboratory Services.

### Clinical assessments, neuropathy and renal disease staging

A comprehensive neurological assessment was conducted in all patients and neuropathy severity was graded according to a modified version of the Total Neuropathy Score (TNS) [19] which has been previously used in a number of large cohort studies in diabetes [12, 20]. The TNS is a composite score of 8 categories which assess extent and severity of sensory and motor symptoms, assessment of deep tendon reflexes, muscle strength, vibration sensibility (128 Hz tuning fork), pinprick sensation (Neurotip^TM^, Owen Mumford, United Kingdom) and tibial and sural nerve amplitudes (Medelec Synergy system, Oxford Instruments, United Kingdom). Each category was scored from 0–4 (0 = no dysfunction, 4 = severe dysfunction) and summed to give a total score from 0–32 (32 = maximum dysfunction). The TNS was further subdivided into total neuropathy grades (TNG); TNG 0 = TNS 0–1, TNG 1 = TNS 2–8, TNG 2 = TNS 9–16, TNG 3 = 17–24 and TNG 4 = TNS 25–32 [19]. Severity of chronic kidney disease (CKD) was quantified in stages according to eGFR. Specifically, CKD stage 1: ≥90, CKD stage 2: 60–89, CKD stage 3: 20–59 and CKD stage 4: <20mL/min/1.73m^2^.

### Assessment of axonal ion channel properties

Axonal excitability studies were undertaken in all patients [21]. These studies involved stimulating the median nerve at the wrist and measuring the compound muscle action potential (CMAP) of abductor pollicis brevis using surface electrodes (Unomedical, Bikerod, Denmark). Parameters of axonal excitability were derived using QTRAC automated software (Digitimer, London, UK) which specifically applied the TRONDNF protocol [22]. QTRAC software allowed the rapid acquisition of a number of excitability parameters which are derived from five distinct testing paradigms; (i) stimulus response behaviour, (ii) strength duration relationship, (iii) threshold electrotonus, (iv) recovery cycle and (v) current threshold relationship.

Stimulus response curves were derived by applying a current of 1ms duration and increasing the intensity until the maximal CMAP response was obtained. Subsequently, a target for the remaining test paradigms was established (~40% of the maximum CMAP). The stimulus intensity required to reach this target was termed “threshold”. Strength-duration-time-constant (SDTC) was obtained by plotting the relationship between stimulus strength and stimulus duration using at least two duration points. This constant was determined to be the ratio of threshold current increase to stimulus duration decrease, and partly reflects the activity of nodal persistent Na^+^ conductances [23], which are thought to underlie the generation of ectopic neuropathic symptoms [24, 25]. Threshold electrotonus was determined by plotting percentage change of threshold when 1ms test impulses were delivered during and after 100ms subthreshold conditioning currents of +40% (TEd) and -40% (TEh) control threshold. This paradigm provides information regarding internodal properties and overall axonal membrane potential [11]. The recovery cycle assesses the recovery of axonal conduction following a supramaximal stimulus. The percentage of threshold change in the recovery cycle was measured by varying the delay between the test impulse (1ms of threshold current) and a conditioning stimulus that was supramaximal. [22]. Current threshold relationship was obtained by plotting the change in threshold with 1ms test impulses post the delivery of 200ms depolarising and hyperpolarising conditioning currents (+50 to– 100ms). This paradigm provides information on the rectifying properties of the internodal region of the axon [11].

### Statistical analysis

SPSS statistics software v.20 (IBM, Chicago, IL) was used for group comparisons. Specifically, to investigate differences in axonal excitability parameters between patient data and age and sex matched normal controls (n = 42), normality of all data was first assessed using the Shapiro Wilk test. Whole group patient data (T2DM) was then compared to normal control data using either an independent t test or Mann Whitney U test depending on the outcome of the normality assessment. To determine axonal excitability alterations in non-neuropathic patients, a subgroup of the T2DM group with no neuropathy (T2DM-NN) was compared to normal control data using either an independent t test or Mann Whitney U test dependent on normality of data. Criteria for inclusion in the T2DM-NN was a TNG score of 0, indicating there were no signs and symptoms of neuropathy in addition to normal nerve conduction study results.

To determine the relationship between triglyceride levels and axonal function, we performed linear regression adjusting for renal function and neuropathy severity in the whole cohort (T2DM). We examined eight parameters of interest which routinely show abnormality in type 2 diabetic patients and progressively alter with the development of DPN [12]: TEh(90–100ms), TEd(10–20ms), SDTC (ms), superexcitability, subexcitability, S2 accommodation, and superexcitability at 7ms. We separately evaluated each parameter for association with triglycerides (TG) using linear regression adjusted for neuropathy TNS and eGFR. To evaluate sensitivity of analyses to categorisation of the confounders, a secondary analysis was conducted adjusting for neuropathy severity (measured by TNS and TNG) and severity of kidney disease (eGFR and CKD stage). 95% confidence intervals were computed for all estimates and p-values <0.05. This analysis was repeated for the low density lipoprotein (LDL) outcome.

## Results

A total of 71 patients with type 2 diabetes were assessed. Patient clinical characteristics are provided in Table 1. Patients mean age was 64.2±2.3 years and had slightly elevated HbA1c% and serum lipids. eGFR indicated on average mild renal impairment in the cohort, denoted by stage 2 (60-89mL/min/1.73m^2^). Total Neuropathy Scores (TNS) from the patients revealed that 42% of patients (n = 51) had clinical signs and symptoms of DPN, which is consistent with previous studies in the literature [26].

**Table 1:** Clinical characteristics of the patient cohort.

	Diabetic patients
	n = 71
Age (years)	64.2±2.3
Gender (M:F)	50:21
HbA1c%	7.8±0.3%
eGFR (mL/min/1.73m^2^)	67.3±5
Triglyceride (mmol/L)	1.6±0.1
Cholesterol (mmol/L)	4.1±0.2
LDL (mmol/L)	2.0±0.2
HDL (mmol/L)	1.3±0.1
TNS (TNG)	5.1±1.3 (2)
CKD stage	2

Clinical characteristics of the patient cohort. Values are given as mean±SE. Units are provided. CKD stages were defined as stage 1: ≥90, 2: 60–89, 3: 20–59 and 4: <20mL/min/1.73m^2^.

### Excitability was abnormal between patients and age matched controls

Median axonal excitability parameters obtained from the patients were compared to age and sex matched normal control data, revealing multiple abnormalities in median axonal excitability properties (Table 2). These were limited primarily to threshold electrotonus and recovery cycle parameters and were consistent with findings in previous studies performed in patients with DPN [12]. Critically, differences were seen in both whole group data taken from the type 2 diabetic patients (T2DM) and in a subset of this patient cohort without signs or symptoms of DPN (T2DM-NN).

**Table 2:** Axonal excitability parameters in patients vs controls.

	*Subjects*		
*Parameter*	*Controls*	*T2DM*	*T2DM-NN*
	*n = 42*	*n = 71*	*n = 20*
*Latency (ms)*	6.9±0.2	7.2±0.1**	7.0±0.2
*Stimulus response*			
*Stimulus for 50% of peak (mA)*	3.5±1.1	4.1±1.1	4.1±0.4
*Stimulus–response slope*	4.5±1.1	4.2±1.0	4.6±0.3
*Strength duration relationship*			
*SDTC (ms)*	0.4	0.5	0.4
*Rheobase (mA)*	2.4±1.1	2.7±1.1	2.7±0.3
*Threshold electrotonus*			
*TEd(10–20ms)*	68.4±0.8	63.9±0.8***	64.5±1.6*
*TEd(peak)*	67.8±0.8	63.3±0.8***	64.2±1.5*
*S2 accommodation*	22.7±0.5	19.2±0.4****	19.7±0.8**
*TEd(undershoot)*	-18.8±0.6	-16.1±0.5**	-14.7±0.9***
*TEh(90–100ms)*	-115.5±2.6	-109.7±2.6	-107.4±3.7
*TEh(overshoot)*	16.7±0.7	13.4±0.5***	12.6±1.0**
*Recovery cycle*			
*Relative refractory period (ms)*	3.2±0.1	3.4±0.1	3.3±0.2
*Superexcitability*	-22.5±0.9	-17.5±0.8***	-17.3±1.6**
*Superexcitability at 5ms*	-22.1±1.0	-16.4±1.1***	-18.3±2.4*
*Superexcitability 7ms*	-21.3±1.0	-16.9±0.7***	-17.6±1.4*
*Subexcitability*	13.8±0.5	11.2±0.5***	11.5±0.7*
*Current threshold relationship*			
*Resting slope*	0.6	0.6	0.6
*Minimum slope*	0.3	0.3	0.3

Excitability parameters from the type 2 diabetic patients compared to matched normal controls. Multiple abnormalities were noted in excitability parameters when comparing whole group data (T2DM) from the patients and controls. A subgroup of patients (n = 20) without clinical signs and symptoms of DPN (T2DM-NN) were also compared to controls revealing multiple abnormalities in axonal excitability. All recovery cycle and threshold electrotonus parameters are expressed as % change in threshold unless otherwise indicated. Significance is indicated by

*P<0.05

**P<0.005

***P<0.0005 and

****P<0.0001.

**Fig 1:**
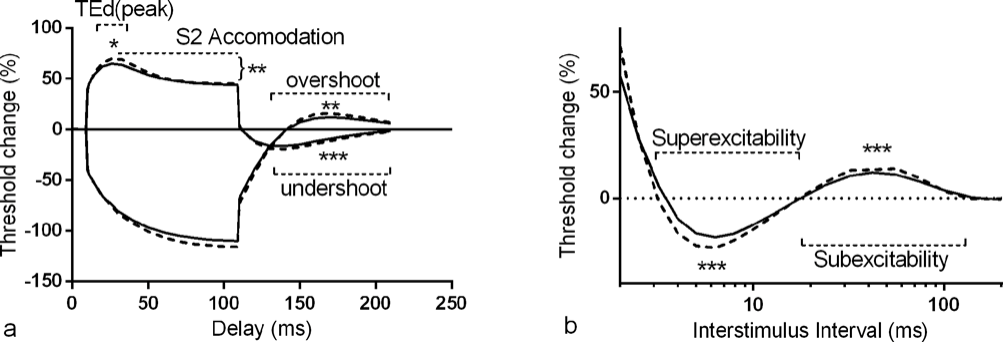
Mean threshold electrotonus and recovery cycle plots from type 2 diabetic patients without neuropathy compared to matched normal controls. Type 2 diabetic patients (block lines) exhibited less threshold change during both threshold electrotonus (a) and the recovery cycle (b) compared to control subjects (dashed lines). Threshold electrotonus parameters are expressed as percentage threshold change during and after subthreshold depolarising and hyperpolarising currents up to 100ms whilst the recovery cycle is given as percentage threshold change at varying intervals after a supramaximal impulse. Significance is indicated by: *P<0.05, **P<0.005 and ***P<0.0005.

Specifically, reduced percentage threshold change was noted during the threshold electrotonus paradigm in response to depolarising (TEd) and hyperpolarising (TEh) currents of varying delays (Table 2 and Fig 1A).

During the recovery cycle paradigm (Table 2 and Fig 1B), reductions were most notable during superexcitability, with corresponding alterations at 5 and 7ms, and subexcitability. Relative refractory periods were not significantly different between controls and either T2DM or T2DM-NN group data. During the strength duration paradigm, SDTC appeared longer in the T2DM group but this was not statistically significant. Cumulatively, the changes noted in threshold electrotonus and recovery cycle parameters are consistent with depolarisation of the axonal membrane potential [14, 27, 28].

### Axonal excitability was not associated with serum triglyceride and LDL

**Fig 2:**
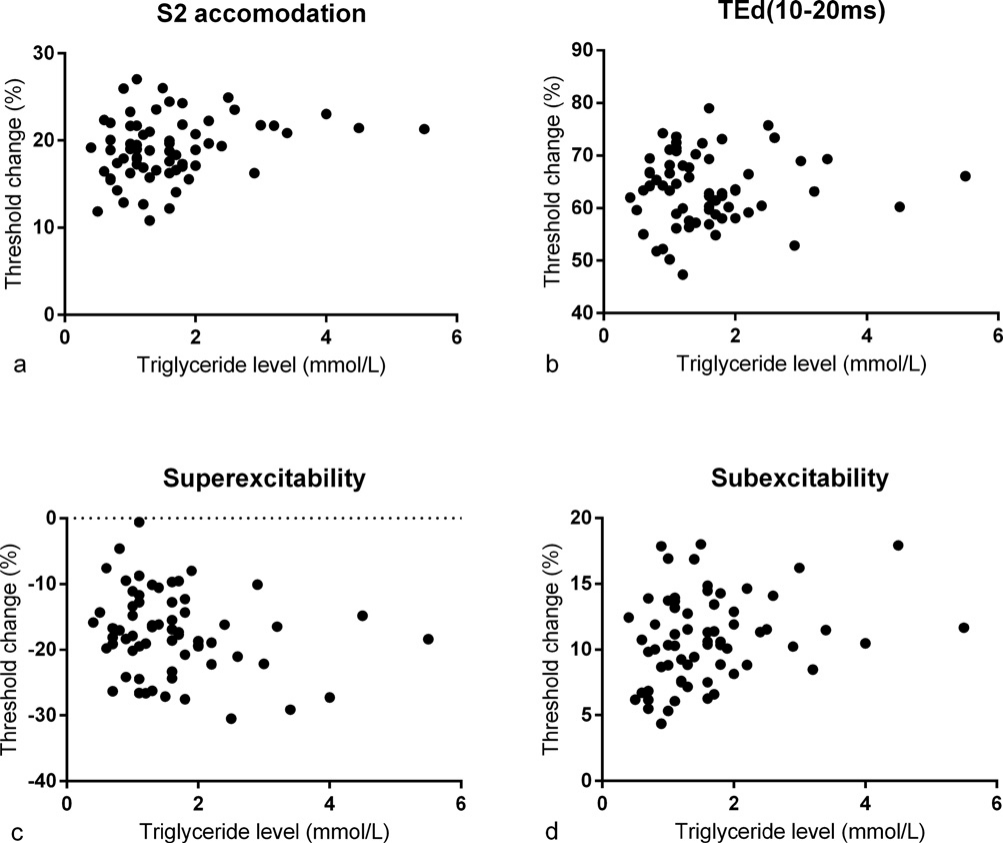
Scatter plots of patient triglyceride levels against excitability parameters. Triglyceride levels are plotted against axonal excitability parameters that are typically abnormal in type 2 diabetic patients: a) S2 accommodation and b) TEd(10–20ms) which are derived from the threshold electrotonus paradigm and provide indication on nodal and internodal ion channel activity; c) superexcitability and d) subexcitability which provide indication of paranodal and nodal K^+^ conductances respectively.

Serum triglyceride and LDL levels were obtained in all 71 participants. Triglycerides ranged from 0.4 to 5.5mmol/L, LDL levels ranged from 0.6 to 4.6mmol/L and eGFR ranged from 26 to 137mL/min/1.73m^2^. There were no associations between axonal excitability parameters and serum triglycerides (Table 3 and Fig 2). Notably, no associations were observed in the sensitivity analysis which was adjusted for neuropathy severity (TNG) and nephropathy severity (CKD stage) (Table 3).

**Table 3:** Linear Regression model between serum triglycerides and excitability parameters typically abnormal in type 2 diabetic patients.

	*Triglycerides*			
*Parameter*	Adjusted*	*p*	*Sensitivityᶺ*	*p*
*SDTC (ms)*	0.01 (-0.03, 0.05)	0.7	0.003 (-0.02, 0.027)	0.83
*TEd(10–20ms)*	1.2 (-1.4, 3.8)	0.38	0.15 (-1.6, 1.9)	0.87
*S2 accommodation*	1.0 (-0.41, 2.4)	0.16	0.94 (0.009, 1.9)	0.05
*TEd(40–60ms)*	0.32 (-1.7, 2.4)	0.76	-0.47 (-1.8, 0.88)	0.49
*TEh(90–100ms)*	3.2 (-6.5, 13)	0.51	2.9 (-3.5, 9.4)	0.36
*Superexcitability*	2.4 (-0.049, 4.8)	0.06	1.3 (-0.41, 2.9)	0.14
*Subexcitability*	0.85 (-0.46, 2.2)	0.2	0.85 (-0.01, 1.7)	0.05
*Superexcitability 7ms*	2.3 (0.11, 4.5)	0.04	1.2 (-0.26, 2.7)	0.10

Linear Regression model between serum triglycerides and excitability parameters typically abnormal in type 2 diabetic patients. Model adjusted for neuropathy (TNS and TNG) and nephropathy severity (eGFR and CKD stage).

*Model adjusted for TNS and eGFR.

ᶺ Model adjusted for TNG and CKD stage.

Similarly, in the analysis of LDL and axonal excitability parameters (Table 4), no notable associations were observed.

**Table 4:** Linear Regression model between serum LDL and excitability parameters typically abnormal in type 2 diabetic patients.

	*LDL*			
*Parameter*	Adjusted*	*p*	*Sensitivityᶺ*	*p*
*SDTC (ms)*	-0.02 (-0.05, 0.02)	0.32	-0.01 (-0.04, 0.015)	0.33
*TEd(10–20ms)*	-0.29 (-2.5, 2)	0.8	0.14 (-1.9, 2.2)	0.89
*S2 accommodation*	-0.46 (-1.7, 0.84)	0.48	0.048 (-1.1, 1.2)	0.94
*TEd(40–60ms)*	0.44 (-1.2, 2.1)	0.59	0.078 (-1.5, 1.6)	0.92
*TEh(90–100ms)*	-0.54 (-9.5, 8.4)	0.9	0.85 (-6.9, 8.6)	0.83
*Superexcitability*	0.12 (-2.1, 2.3)	0.91	0.57 (-1.5, 2.7)	0.58
*Subexcitability*	-0.007 (-1.2, 1.1)	0.99	0.37 (-0.68, 1.4)	0.48
*Superexcitability 7ms*	0.53 (-1.5, 2.5)	0.59	0.55 (-1.3, 2.4)	0.55

Linear Regression model between serum LDL and excitability parameters typically abnormal in type 2 diabetic patients. Model adjusted for neuropathy (TNS and TNG) and nephropathy severity (eGFR and CKD stage).

*Model adjusted for TNS and eGFR.

ᶺ Model adjusted for TNG and CKD stage.

## Discussion

The present study was undertaken to prospectively evaluate the association between serum triglyceride and LDL levels and changes in axonal function in a type 2 diabetic cohort. Statistical analyses were undertaken and adjusted for confounders (neuropathy severity and renal function) which have previously been associated with alterations in axonal function in a T2DM cohort [12, 29, 30]. Our study indicates that after accounting for these variables, there were no significant associations between altered serum triglyceride or LDL concentration and changes in axonal function in T2DM patients. It should be noted that while these studies were undertaken prospectively, a significant percentage of the cohort had serum triglyceride levels that were within the normal range and previous work has suggested that the neurotoxic effects are mediated by hypertriglyceridemia [31], which may explain the lack of association in the present study. A limitation of the study was the lack of stratification for duration of diabetes which is a risk factor for neuropathy development [1]. Furthermore, we did not stratify for total cholesterol levels or the duration of their presence. This is of potential importance as both low HDL and opposing high LDL cholesterol levels have been previously associated with neuropathy development [32].

The pattern of change in axonal function noted in the present study is consistent with previous investigations undertaken in type 2 diabetic patients [12,14,17,26]. Given that these changes were evident in the absence of an association with serum lipids, it would appear from the present investigation that abnormalities of axonal function are not related to serum triglyceride levels in type 2 diabetic patients. Moreover, although a previous study in a smaller cohort suggested that triglyceride levels were associated with axonal dysfunction [15], the present study as well as the previous study assessed patients at a single time point and it is possible that a large cumulative level of exposure to elevated triglyceride concentrations is required before alterations in axonal function occur.

Previous clinical studies have indicated that dyslipidemia may represent a risk factor for neuropathy development in T2DM [33, 34]. Companion studies *in vitro* have invoked the possibility that hyperlipidemia in conjunction with hyperglycaemia may potentiate oxidative stress in dorsal root ganglion neurons, leading to mitochondrial injury and axonal degeneration [35–37]. However, in the present study, axonal function was assessed using axonal excitability techniques, which provide information on axonal biophysical properties at the site of stimulation [38], namely the nerve trunk. Excitability techniques do not assess proximal sensory and motor pathways and it is therefore possible that the lack of association suggests that the effects of hyperlipidemia are maximal at the level of the cell body, rather than the nerve trunk. Moreover, previous studies have also suggested that aberrant Schwann cell physiology may exacerbate the development of neuropathy [39, 40] and this may not necessarily be reflected in altered axonal excitability parameters, which largely reflect properties of the axon itself rather than the myelin sheath. The lack of association also suggests that it is unlikely that acutely serum triglycerides have a direct effect on axonal biophysical properties, unlike other potential causes of axonal injury such as severe hyperkalaemia [29] or limb ischaemia [27], which are thought to directly impair axonal function by altering axonal biophysical properties [27].

It is likely that other mediating factors apart from hypertriglyceridaemia and hyperglycaemia may be involved in mediating neuropathy development in type 2 diabetes. Potential candidates include increased circulating inflammatory markers including NF-κB, which is upregulated in sural nerves in patients and rodent models [41–43] and involved in altered thermonociception [42]. Furthermore, the pro-inflammatory cytokine TNF-α has been implicated in mechanical and thermal hyperalgesia in addition to ectopic firing of sensory neurons [41, 44, 45], which occurs in a dose-dependent manner [41]. However a recent human study indicated no correlation between TNF-α and nerve conduction velocities in type 2 diabetic patients [46]. Additionally, downstream mitochondrial dysfunction may also play a role in axonal dysfunction and has been proposed as an additive factor in diabetic neuropathy development [47]. Involved in appropriate cellular respiration, depolarisation of the mitochondrial membrane potential has been observed in DRG sensory neurons in animal models of diabetes and is prevented by insulin administration [48]. From a clinical perspective, the present study therefore suggests that treatment of triglyceride levels alone is unlikely to have any significant impact on axonal properties in T2DM. As axonal function may be involved in neuropathy progression [12], the findings suggest that preventing progression of neuropathy in T2DM may require investigation of other biochemical pathways. While elevated triglyceride levels may still be implicated in the development of small fibre neuropathy, future studies may have to explore other potential avenues of treatment such as control of inflammatory mediators in order to prevent progression of large fibre neuropathy in T2DM.

In summary, the present prospective study has demonstrated no association between changes in axonal function and triglyceride levels in a cohort of type 2 diabetic patients. Furthermore, prospective studies should consider evaluating additional factors in type 2 diabetic patients which may promote diabetic neuropathy, including the role of inflammatory markers which may mediate mitochondrial dysfunction.
